# The Predictive Value of NLR, MLR, and PLR in the Outcome of End-Stage Kidney Disease Patients

**DOI:** 10.3390/biomedicines10061272

**Published:** 2022-05-29

**Authors:** Adrian Vasile Mureșan, Eliza Russu, Emil Marian Arbănași, Réka Kaller, Ioan Hosu, Eliza Mihaela Arbănași, Septimiu Toader Voidăzan

**Affiliations:** 1 Clinic of Vascular Surgery, Mureș County Emergency Hospital, 540136 Târgu Mureș, Romania; adrian.muresan@umfst.ro (A.V.M.); eliza.russu@umfst.ro (E.R.); 2 Department of Surgery, George Emil Palade University of Medicine, Pharmacy, Science, and Technology of Targu Mures, 540139 Târgu Mureș, Romania; 3 Department of Nephrology, Mureș County Emergency Hospital, 540136 Târgu Mureș, Romania; ioanhosu68@yahoo.com; 4 Faculty of Pharmacy, George Emil Palade University of Medicine, Pharmacy, Science, and Technology of Targu Mures, 540139 Târgu Mureș, Romania; arbanasi.eliza@gmail.com; 5 Department of Epidemiology, George Emil Palade University of Medicine, Pharmacy, Science, and Technology of Targu Mures, 540139 Târgu Mureș, Romania; septimiu.voidazan@umfst.ro

**Keywords:** chronic kidney disease, ESKD, NLR, PLR, MLR

## Abstract

Background: Chronic kidney disease (CKD) is a global public health problem with a high mortality rate and a rapid progression to end-stage kidney disease (ESKD). Recently, the role of inflammation and the correlation between inflammatory markers and CKD progression have been studied. This study aimed to analyze the predictive value of the neutrophil–lymphocyte ratio (NLR), monocyte-to-lymphocyte ratio (MLR), and platelet-to-lymphocyte ratio (PLR) in assessing the outcome of ESKD patients. Methods: A retrospective study which included all patients admitted in the Department of Nephrology of the County Emergency Clinical Hospital, Târgu-Mureș, Romania, between January 2016 and December 2019, diagnosed with ESKD. Results: Mortality at 30 days was clearly higher in the case of the patients in the high-NLR groups (40.12% vs. 1.97%; *p* < 0.0001), high-MLR (32.35% vs. 4.81%; *p* < 0.0001), and respectively high-PLR (25.54% vs. 7.94%; *p* < 0.0001). There was also a significant increase in the number of hospital days and the average number of dialysis sessions in patients with high-NLR (*p* < 0.0001), high-MLR (*p* < 0.0001), and high-PLR (*p* < 0.0001). The multivariate analysis showed that a high baseline value for NLR (*p* < 0.0001), MLR (*p* < 0.0001), and PLR (*p* < 0.0001) was an independent predictor of 30-day mortality for all recruited patients. Conclusions: Our findings established that NLR, MLR, and PLR determined at hospital admission had a strong predictive capacity of all-cause 30-day mortality in ESKD patients who required RRT for at least 6 months. Elevated values of the ratios were also associated with longer hospital stays and more dialysis sessions per patient.

## 1. Introduction

Chronic kidney disease (CKD) has become a global public health problem, with rising incidence, high costs, and a high morbidity and mortality rate [1]. According to World Health Organization reports, chronic kidney disease ranks 10th in the world as a cause of death, with 1.3 million deaths reported by 2019 and a significant increase in the last 20 years. In 2000, only 813,000 deaths were reported, and kidney disease ranked 13th worldwide as a cause of death [2].

At the European level, according to reports made by the European Renal Association-European Dialysis and Transplant Association (ERA-EDTA), in 2019, the incidence of patients accepted for Renal Replacement Therapy (RRT) was 132 per million population (pmp). In Romania, in the same period, an incidence of 191 pmp was registered, our country thus occupying seventh place at the European level [3]. At the national level, until 31 December 2020, 13,663 patients were registered for RRT, out of which 13,374 were on hemodialysis, and only 289 were on peritoneal dialysis [4]. The most stable site of dialysis with a long-life expectancy are arteriovenous fistula (AVF) [5], whereas acute ill patients need central venous hemodialysis catheter (CVC) dialysis.

Chronic kidney disease is defined as the presence of renal injury or a decrease of at least three months in the glomerular filtration rate (GFR) below 60 mL/min/1.73 m^2^. It is classified according to the glomerular filtration rate in five stages. The last stage of chronic kidney disease (ESKD) occurs at a GFR < 15 mL/min/1.73 m^2^ and requires RRT [6]. Numerous factors are involved in the degradation of renal function and implicitly in the evolution of CKD, such as hypertension, dyslipidemia, diabetes, old age, and male sex [7,8,9]. Recently, the role of inflammation and the correlation between inflammatory markers and CKD progression have been studied [10,11,12].

One of the most statistically significant inflammatory biomarkers in many fields is the neutrophil–lymphocyte ratio, whose prognostic role has been demonstrated in cardiovascular disease, vascular and oncological surgery (especially gastric, colorectal, prostate, ovarian), and neurology [13,14,15,16,17,18,19,20,21,22,23,24,25,26,27,28].

Numerous studies have recently aimed to analyze the correlation between neutrophil–lymphocyte ratio (NLR) values and the progression of patients with CKD in the initial stages. In the article published by Yuan et al., a prospective study was performed in which 938 patients diagnosed with CKD stages I–IV were included. It was found that the group of patients with NLR ≥ 2.09 had a statistically significant number of progressions to ESKD (92 vs. 31, *p* < 0.0001) [29].

This study aimed to analyze the predictive value of the NLR, monocyte-to-lymphocyte ratio (MLR), and platelet-to-lymphocyte ratio (PLR), and also the outcome of patients with ESKD requiring RRT for at least 6 months, admitted in the Nephrology Department of the Târgu-Mureș County Emergency Clinical Hospital, Romania, between January 2016 and December 2019.

## 2. Materials and Methods

### 2.1. Study Design

This is a retrospective study which included all patients admitted in the Department of Nephrology of the County Emergency Clinical Hospital, Târgu-Mureș, Romania, between January 2016 and December 2019, diagnosed with ESKD, who required RRT for at least 6 months and who had RRT performed via an arteriovenous fistula (AVF) or through a central venous hemodialysis catheter (CVC). Exclusion criteria were CKD stages I–IV, ESKD and peritoneal dialysis, patients diagnosed with systemic inflammatory disease, recent tumor status, hematological disease, personal history of major surgery in the last 6 months, and autoimmune diseases. The patients included in the study were initially divided into two groups based on the poor outcome: patients who survived and those who died. Subsequently, the 30-day death rate, the number of hospital days, and the number of dialysis treatments for each patient were determined based on the ideal cut-off value for NLR, MLR, and PLR versus mortality. The receiver operating characteristic (ROC) curve analysis was used to identify the optimal cut-off values of NLR and PLR according to the Youden index (Youden Index = Sensitivity + Specificity − 1, range from 0 to 1).

### 2.2. Data Collection

From the hospital’s electronic database, the patients’ demographic data were extracted, along with the number of days of hospitalization, the number of dialysis sessions performed during admission, and the type of vascular access for dialysis. The following comorbidities were extracted from the medical history: arterial hypertension (AH), atrial fibrillation (AF), chronic heart failure (CHF), ischemic heart disease (IHD), myocardial infarction (MI), type 2 diabetes (T2D), chronic obstructive pulmonary disease (COPD), cerebrovascular accident (CVA), peripheral artery disease (PAD), dyslipidemia, tobacco usage, and obesity. In the first 24 h after admission, each patient was given a complete set of blood tests (glucose level, hemoglobin, hematocrit, neutrophil count, lymphocyte count, platelet count, serum creatinine, blood urea nitrogen (BUN), and potassium). The neutrophil–lymphocyte ratio was calculated by dividing the total number of neutrophils by the total number of lymphocytes.

### 2.3. Study Outcomes

The primary endpoint was verifying the predictive role of NLR, MLR, and PLR for mortality at 30 days in the case of ESKD patients who were on hemodialysis for at least 6 months. The secondary was the 30-day mortality rate for the patients who had hemodialysis performed via an AVF, respectively, those who had it performed via a CVC. Additionally, the number of hospital days and the number of dialysis sessions per patient were calculated for all the patients, respectively, for the analyzed subclasses.

### 2.4. Ethical Approval

The study was conducted in accordance with the Declaration of Helsinki and approved by the Ethics Committee of Târgu-Mureș Emergency County Hospital, Romania (protocol code 29290, on 10 November 2021). All patients enrolled in the study gave their informed written consent to be included in the present analysis.

### 2.5. Statistical Analysis

Statistical analysis was performed using SPSS for Windows version 28.0.1.0 (SPSS, Inc., Chicago, IL, USA). The associations of NLR, MLR, PLR with category variables were assessed using chi-square tests, while differences in continuous variables were analyzed using t-Student or Mann Whitney tests. The receiver–operating characteristic (ROC) curve analysis was used to test the predictive power and to determine cut-off values of NLR, MLR, and PLR. All tests were two-tailed tests and a *p*-value < 0.05 was considered as statistically significant.

## 3. Results

**Figure 1:**
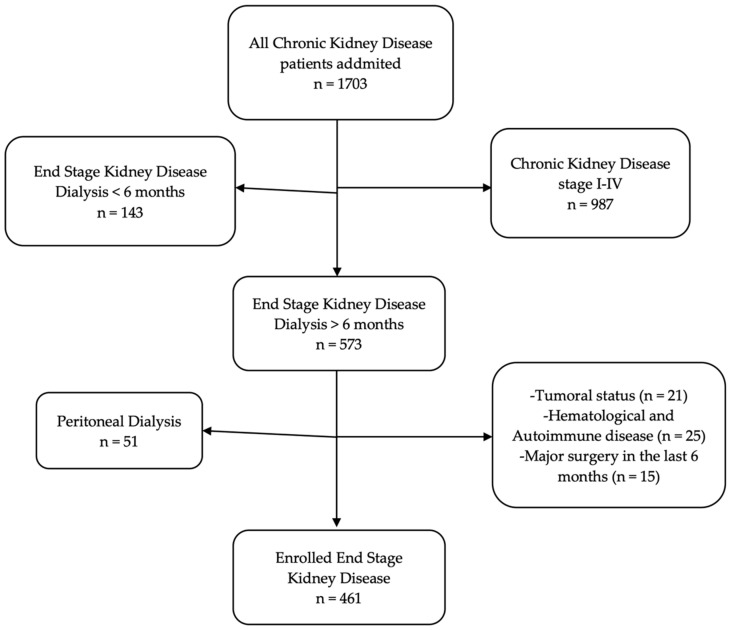
Enrollment flowchart.

During the study period, 1703 patients with CKD were admitted. Of these, 987 patients were CKD stage I-IV, 143 were ESKD with less than 6 months of RRT, 51 patients with PD, 21 had tumoral status, 25 had autoimmune or hematological diseases, and 15 had major surgery in the previous 6 months. Four hundred sixty-one patients with ESKD who met all the criteria were included in the study (Figure 1).

For the whole group, there was an average age of 64.36 ± 12.14, with ages between 19 and 98 years, and a predominance of males (60.52%). Of the group, 191 patients (41.43%) underwent dialysis via an AVF, and 270 (58.57%) through a dialysis CVC. The comorbidities with the highest incidence were AH in 388 patients (84.16%), followed by IHD in 303 patients (65.72%), and CHF in 213 patients (46.2%). As risk factors, dyslipidemia was encountered in 217 patients (47.07%), tobacco usage in 199 patients (43.16%), and obesity in 139 patients (30.15%). Regarding the number of days of admission, an average of six days of hospitalization and four dialysis sessions per patient were observed. At 30 days, there was a mortality rate of 14.96% (Table 1).

Depending on the mortality at 30 days, the patients were divided into two groups. The first group included those with a positive outcome at 30 days, and in the second group were patients with poor outcomes at 30 days. Distribution by sex, mean age, comorbidities, type of vascular access, and laboratory data are presented in Table 2 and Table 3.

Significant differences were found in the average age of the patients, with a higher value in patients with poor outcomes (68.82 vs. 63.58; *p* = 0.001). Regarding comorbidities, patients with poor outcomes have a higher incidence of AF (34.78% vs. 18.11%; *p* = 0.002), MI (40.57% vs. 17.6%; *p* < 0.0001) and CVA (36.23% vs. 23.46% *p* = 0.02). Among the risk factors, smoking has a higher incidence in patients with poor outcomes (66.67% vs. 39.03%; *p* < 0.0001). In addition, in the case of patients with a positive outcome, we have a higher incidence of AH (86.98% vs. 68.11%; *p* = 0.0001).

Regarding the laboratory data, in the group with poor outcomes, we found an almost double average value of the total number of neutrophils (11.29 vs. 6.45; *p* < 0.0001), a lower average value of the total number of lymphocytes (0.62 vs. 1.31; *p* < 0.0001) and of the total platelet count (PLT) (172 vs. 216.95; *p* < 0.0001) were found. Moreover, a higher average value of NLR (*p* < 0.0001), MLR (*p* < 0.0001), and PLR (*p* < 0.0001) was recorded in patients with poor outcomes.

**Figure 2:**
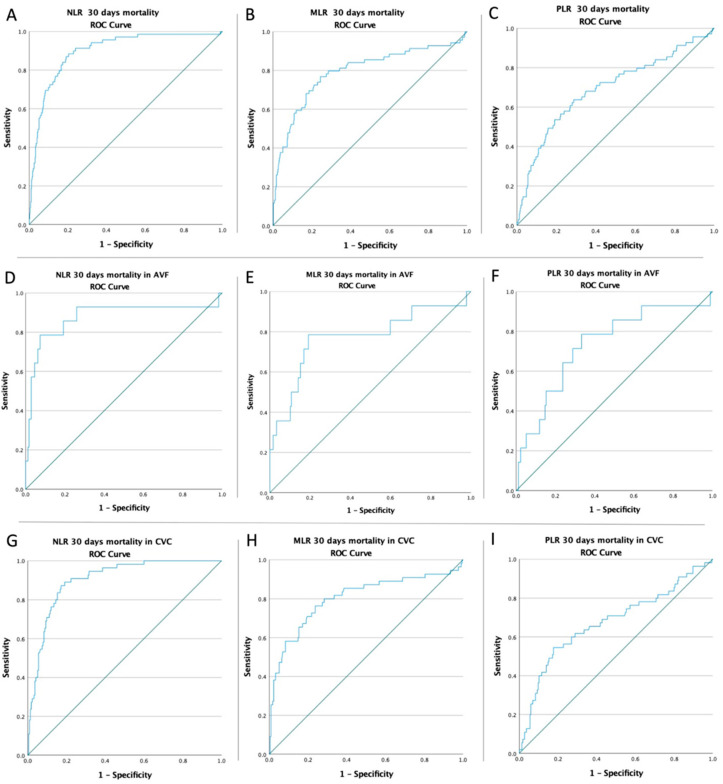
ROC curve analysis (**A**) for NLR concerning the mortality rate in all patients (AUC = 0.897; *p* < 0.0001), (**B**) for MLR concerning mortality in all patients (AUC = 0.792; *p* < 0.0001), (**C**) for PLR concerning mortality rate in all patients (AUC = 0.692; *p* < 0.0001), (**D**) for NLR concerning mortality in AVF patients (AUC = 0.837; *p* < 0.0001), (**E**) for MLR concerning mortality in AVF patients (AUC = 0.753; *p* = 0.001), (**F**) for PLR concerning mortality in AVF patients (AUC = 0.709; *p* = 0.008), (**G**) for NLR concerning the mortality rate in CVC patients (AUC = 0.887; *p* < 0.0001), (**H**) for MLR concerning the mortality rate in CVC patients (AUC = 0.783; *p* < 0.0001), (**I**) for PLR concerning the mortality rate in CVC patients (AUC = 0.674; *p* < 0.0001).

Receiver operating characteristic (ROC) curves of NLR, MLR, and PLR were created to determine whether the baseline of these biomarkers was predictive for mortality in all patients, mortality in AVF patients, and mortality in CVC patients (Figure 2). The optimal cut-off obtained from Youden’s index, areas under the curve (AUC), and the predictive accuracy of inflammatory markers are listed in Table 4.

Depending on the optimal cut-off value, according to ROC, for NLR (8.19), MLR (0.63), and PLR (199.05), the patients included in the study were divided into two groups, and the outcome was studied. Mortality at 30 days was clearly higher in the case of the patients in the high-NLR groups (40.12% vs. 1.97%; *p* < 0.0001), high-MLR (32.35% vs. 4.81%; *p* < 0.0001), and respectively high-PLR (25.54% vs. 7.94%; *p* < 0.0001). There was also a significant increase in the number of hospital days and the average number of dialysis sessions in patients with high-NLR (*p* < 0.0001), high-MLR (*p* < 0.0001), and high-PLR (*p* < 0.0001). (Table 5).

The multivariate analysis showed that a high baseline value for NLR, MLR, and PLR was an independent predictor of 30 days mortality for all recruited patients. Furthermore, for all hospitalized patients AF, MI, and tobacco use was an independent predictor of short time mortality. In terms of type of vascular access, AVF acted as a protective factor against mortality. However, CVC was an independent predictor of mortality (Table 6).

## 4. Discussion

This study included 461 patients. A comprehensive study was carried out to determine the predictive role of NLR, MLR, and PLR values in assessing the poor outcome of patients with ESKD who had been on hemodialysis for at least 6 months. It turned out that all three factors had an independent role in predicting mortality at 30 days, the highest accuracy belonging to NLR, with a sensitivity of 91.3% and a specificity of 76%, and an AUC of 0.897. In addition, the predictability of inflammatory biomarkers was validated by the 30-day mortality in patients undergoing dialysis via an AVF and by the 30-day mortality in patients with a CVC. All three ratios proved to have a predictive role in these cases, with NLR having the greatest accuracy.

The pro-inflammatory role of neutrophils and lymphocytes in immune system regulation is well known [30,31]. Systemic inflammation has been associated with increases in lymphocyte apoptosis [32], as well as an increased risk of infection [33] and unfavorable cardiovascular events [34]. Elevated NLR values are based on increased neutrophil counts and decreased lymphocyte counts and reflect the balance between systemic inflammation and immune response. In addition to their known hemostatic role, platelets play an essential role in the inflammatory process and immunological responses [35,36,37].

NLR and MLR are measures of acute myeloid-driven innate immune responses reported to chronic, lymphocyte-driven, immunological memory reflected by lymphocyte numbers. An increased MLR and NLR may reflect an immunological imbalance between a potential ongoing clinical or sub-clinical acute inflammation and an impaired immune defense against pathogens.

The paper published by Reddan et al., which included 25,661 hemodialysis patients from 1998 facilities for chronic dialysis, highlighted the negative outcome of high neutrophil counts and low lymphocytes counts [38]. As shown in Table 3, the group of patients with a poor outcome had a significant increase in the number of neutrophils (11.29 vs. 6.45; *p* < 0.0001), respectively a decrease in the number of lymphocytes (0.62 vs. 1.31; *p* < 0.0001) and platelets (172 vs. 216.95; *p* < 0.0001). Regarding the ratios, in the case of deceased patients, there was an increased average value for NLR (17.91 vs. 4.63; *p* < 0.0001), MLR (1 vs. 0.45; *p* < 0.0001), and PLR (273.25 vs. 163.57; *p* < 0.0001).

CKD is a global public health problem with a high mortality rate and a rapid progression to ESKD. In the study by Kim et al., the mortality rate in patients with CKD (63.9%) was higher than in the group of patients with AH or T2D (24.9%) or in the control group (20.8%) [39]. The establishment of the predictive role of the biomarkers for both the progression of CKD and the negative outcome is an intensely discussed topic in recent years’ literature. High NLR and PLR values have been associated with the evolution of CKD towards ESKD and with a high mortality rate in numerous studies [29,40,41,42,43,44,45,46,47].

The predictive role of NLR on the mortality of ESKD patients on dialysis has been demonstrated in several recently published papers [46,47,48,49,50,51,52,53,54,55]. Some of these papers compared the values of NLR and PLR. Thus, Catabay et al. conducted a study that included 108 548 patients and reported the connection between high NLR values and high mortality rates, compared to PLR values [48]. Yoshitomi et al. demonstrated that a high NLR was associated with poor renal outcome (HR 1.67, 95% CI 1.02–2.77) in 350 patients with stage I-IV CKD [49]. Similarly, the article published by Zhang et al. showed the association of high NLR values with all causes of mortality. In contrast, high PLR values are a predictive factor for cardiovascular mortality [44]. In the paper published by Xiang et al., which included 355 patients on dialysis for at least 6 months, high MLR values were found to be independent factors of adverse prognosis [56].

Regarding the predictive role of PLR, Yaprak et al. demonstrated the superiority of PLR’s capacity to predict mortality compared to NLR in the adjusted model [57]. Moreover, the recent paper of Brito et al. showed that the PLR, but not the NLR, was positively correlated with hs-CRP in nondialysis CKD patients (*p* = 0.015) [47]. Furthermore, in the meta-analysis published by Ao et al., showed that a high NLR is related to all-cause mortality (HR 1.93, 95% CI 1.87–2.00; *p* < 0.0001), and cardiovascular mortality (HR 1.45, 95% CI 1.18–1.79; *p* < 0.001) in 116,709 patients with CKD [58]. Additionally, similarly to the previously meta-analysis, Zhao et al., demonstrated that a high NLR predict all-cause mortality and cardiovascular events in 1442 patients with CKD [59].

The discovery and validation of these predictive biomarkers on the negative outcome of these frail hemodialysis patients is a critical point in improving their quality of life and increasing their life expectancy. According to the results of this study and the recent literature, the values of NLR, MLR, and PLR are independent factors in predicting the negative outcome of patients with ESKD.

To our knowledge, following the search in the leading medical databases (PubMed, Embase, Google Scholar, Scopus, Web of Science), this study is the first to verify the association between high values of NLR, MLR, and PLR and the mortality rate, hospital stay and number of dialysis sessions of patients on hemodialysis for at least 6 months.

Our study has some limitations, including the retrospective approach, with a limited number of patients from a single center. Carrying out a prospective, randomized study, including a control group and monitoring the long-term outcome is recommended in the future. Another limitation is excluding patients with peritoneal dialysis, the group of patients with the highest risk of adverse events, in order to achieve uniformity in methodology, and avoid questionable results. Moreover, we could not adjust the models to the immunosuppression timing, bone marrow status, other medication, day of dialysis, sampling site, the potential presence of infection, or bone marrow status. Unfortunately, data on these parameters, which might have influenced the blood cell analysis and outcome, were not available from this cohort.

## 5. Conclusions

Our findings established that NLR, MLR, and PLR determined at hospital admission had a strong predictive capacity of all-cause 30-day mortality in ESKD patients who required RRT for at least 6 months. Elevated values of the ratios were also associated with longer hospital stays and more dialysis sessions per patient. Given the accessibility and low cost of the ratios, future research should investigate means to reduce these biomarkers values, in order to improve these patients’ outcome and quality of life.

## Figures and Tables

**Table 1:** Demographic, comorbidities, risk factors, type of dialysis access, and outcome of all patients included in the analysis.

Variables	All Patients *n* = 461
Age mean ± SD (min–max)	64.36 ± 12.14 (19–98)
Male sex no. (%)	279 (60.52%)
Comorbidities and Risk factors	
AH, no. (%)	388 (84.16%)
AF, no. (%)	95 (20.6%)
CHF, no. (%)	213 (46.2%)
IHD, no. (%)	303 (65.72%)
MI, no. (%)	97 (21.04%)
T2D, no. (%)	164 (35.57%)
COPD, no. (%)	133 (28.85%)
CVA, no. (%)	117 (25.37%)
PAD, no. (%)	143 (31.01%)
Tobacco, no. (%)	199 (43.16%)
Obesity, no. (%)	139 (30.15%)
Dyslipidemia, no. (%)	217 (47.07%)
Type of dialysis access	
AVF, no. (%)	191 (41.43%)
CVC, no. (%)	270 (58.56%)
outcomes	
Hospital stay, day median [Q1–Q3]	6 [4–11]
Dialysis session on patient, no. median [Q1–Q3]	4 [2–6]
30-day mortality, no. (%)	69 (14.96%)

AH = arterial hypertension; AF = atrial fibrillation; CHF = chronic heart failure; IHD = ischemic heart disease; MI = myocardial infarction; T2D = type 2 diabetes; COPD = chronic obstructive pulmonary disease; CVA = cerebrovascular accident; PAD = peripheral artery disease; AVF = arteriovenous fistula; CVC = central venous catheter; SD = standard deviation.

**Table 2:** Demographic, comorbidities, risk factors, type of dialysis access of the two sub-groups evaluated according to the poor outcome.

	Survivors *n* = 392	Deaths *n* = 69	*p* Value (OR; CI 95%)
Age mean ± SD (min–max)	63.58 ± 12.04 (19–89)	68.82 ± 11.83 (46–98)	0.001 ^#^
Male sex no. (%)	234 (59.69%)	45 (65.21%)	0.38 ^¥^ (0.79; 0.46–1.34)
Comorbidities and Risk factors			
AH, no. (%)	341 (86.98%)	47 (68.11%)	0.0001 ^¥^ (3.12; 1.74–5.62)
AF, no. (%)	71 (18.11%)	24 (34.78%)	0.002 ^¥^ (0.41; 0.23–0.72)
CHF, no. (%)	176 (44.89%)	37 (53.62%)	0.18 ^¥^ (0.7; 0.42–1.17)
IHD, no. (%)	261 (66.58%)	42 (60.86%)	0.35 ^¥^ (1.28; 0.75–2.16)
MI, no. (%)	69 (17.6%)	28 (40.57%)	<0.0001 ^¥^ (0.31; 0.18–0.54)
T2D, no. (%)	144 (36.73%)	20 (28.98%)	0.21 ^¥^ (0.81; 0.7–2.48)
COPD, no. (%)	109 (27.8%)	24 (34.78%)	0.23 ^¥^ (0.72; 0.41–1.24)
CVA, no. (%)	92 (23.46%)	25 (36.23%)	0.02 ^¥^ (0.53; 0.31–0.92)
PAD, no. (%)	118 (30.1%)	25 (36.23%)	0.31 ^¥^ (0.75; 0.44–1.29)
Tobacco, no. (%)	153 (39.03%)	46 (66.67%)	<0.0001 ^¥^ (0.32; 0.18–0.54)
Obesity, no. (%)	119 (30.35%)	20 (28.98%)	0.81 ^¥^ (1.06; 0.6–1.87)
Dyslipidemia, no. (%)	183 (46.68%)	35 (49.27%)	0.69 ^¥^ (0.9; 0.54–1.5)
Type of dialysis access			
AVF, no. (%)	177 (45.15%)	14 (20.28%)	0.01 ^¥^ (0.46; 0.25–0.87)
CVC, no. (%)	215 (54.84%)	55 (79.71%)	

AH = arterial hypertension; AF = atrial fibrillation; CHF = chronic heart failure; IHD = ischemic heart disease; MI = myocardial infarction; T2D = type 2 diabetes; COPD = chronic obstructive pulmonary disease; CVA = cerebrovascular accident; PAD = peripheral artery disease; AVF = arteriovenous fistula; CVC = central venous catheter; SD = standard deviation; ^#^, student *t* test; ^¥^, chi square test.

**Table 3:** Laboratory data of the two sub-groups evaluated according to poor outcome.

	Survivors *n *= 392	Deaths *n* = 69	*p* Value ^§^
Haemoglobin g/dL median [Q1–Q3]	9.81 [8.41–11.3]	9.6 [8.2–11.3]	0.28
Haematocrit % median [Q1–Q3]	30.76 [26.2–34.4]	31.19 [25.3–35.72]	0.41
Neutrophils ×10³/uL median [Q1–Q3]	6.45 [4.57–8.25]	11.29 [8.49–14.8]	<0.0001
Lymphocytes ×10³/uL median [Q1–Q3]	1.31 [0.94–1.83]	0.62 [0.4–1.0]	<0.0001
Monocyte ×10³/uL median [Q1–Q3]	0.62 [0.45–0.88]	0.64 [0.46–1.0]	0.24
PLT ×10³/uL median [Q1–Q3]	216.95 [170.17–272.0]	172 [125–235]	<0.0001
Glucose mg/dL median [Q1–Q3]	112 [95.0–143.25]	116 [93–165]	0.23
BUN mg/dL median [Q1–Q3]	137.5 [99.45–191.9]	141.3 [98.95–220.1]	0.2
Creatinine mg/dL median [Q1–Q3]	7.45 [5.87–9.63]	7.41 [6.29–9.74]	0.29
Potassium mmol/L median [Q1–Q3]	5.2 [4.65–6.06]	5.1 [4.5–6.48]	0.35
NLR median [Q1–Q3]	4.63 [2.87–7.89]	17.91 [11.53–24.54]	<0.0001
MLR median [Q1–Q3]	0.45 [0.32–0.68]	1 [0.7–1.63]	<0.0001
PLR median [Q1–Q3]	163.57 [115.22–238.23]	273.25 [161.87–411.11]	<0.0001

PLT = total platelet count; BUN = blood urea nitrogen; NLR = neutrophil–lymphocyte ratio; MLR = monocyte-to-lymphocyte ratio; PLR = platelet-to-lymphocyte ratio; ^§^, Mann Whitney test.

**Table 4:** ROC curves, optimal Cut-Off value, AUC, and predictive accuracy of inflammatory markers NLR, MLR, and PLR.

Variable	Cut-Off	AUC	Std. Error	95% CI	Sensitivity	Specificity	*p* Value
Mortality rate in all patients							
NLR	8.19	0.897	0.020	0.857–0.937	91.3%	76%	<0.0001
MLR	0.63	0.792	0.035	0.724–0.860	79.7%	71.4%	<0.0001
PLR	199.05	0.692	0.038	0.617–0.767	68.1%	65.1%	<0.0001
Mortality rate in AVF patients							
NLR	13.78	0.875	0.067	0.743–1.000	78.6%	92.7%	<0.0001
MLR	0.809	0.772	0.080	0.615–0.928	78.6%	80.8%	0.001
PLR	198.19	0.734	0.074	0.589–0.879	78.6%	67%	0.004
Mortality rate in CVC patients							
NLR	8.07	0.902	0.020	0.862–0.942	90.9%	77.7%	<0.0001
MLR	0.69	0.802	0.039	0.725–0.879	76.4%	76.3%	<0.0001
PLR	224.46	0.675	0.045	0.587–0.763	61.8%	71.2%	<0.0001

AUC = areas under the curve; CI = confidence interval.

**Table 5:** Outcomes of the two sub-groups evaluated separately according to the optimal cut-off value of NLR, MLR, and PLR.

	Low-NLR *n* = 304	High-NLR *n* = 157	*p* Value
Hospital stay, day median [Q1–Q3]	5 [4–9]	10 [6–14]	<0.0001 ^§^
Dialysis session on patient, no. median [Q1–Q3]	2 [3–5]	5 [3–7]	<0.0001 ^§^
30-day mortality, no. (%)	6 (1.97%)	63 (40.12%)	<0.0001 ^¥^
	low-MLR *n* = 291	high-MLR *n* = 170	*p* value
Hospital stay, day median [Q1–Q3]	5 [3.5–9]	8 [5–12]	<0.0001 ^§^
Dialysis session on patient, no. median [Q1–Q3]	3 [2–5]	4 [3–7]	<0.0001 ^§^
30-day mortality, no. (%)	14 (4.81%)	55 (32.35%)	<0.0001 ^¥^
	low-PLR *n* = 277	high-PLR *n* = 184	*p* value
Hospital stay, day median [Q1–Q3]	5 [3–9]	9 [5–12]	<0.0001 ^§^
Dialysis session on patient, no. median [Q1–Q3]	3 [2–5]	5 [3–7]	<0.0001 ^§^
30-day mortality, no. (%)	22 (7.94%)	47 (25.54%)	<0.0001 ^¥^

OR = odd ratio; ^§^, Mann Whitney test; ^¥^, chi square test.

**Table 6:** Multivariate analysis on 30 days mortality occurrences during the entire study period.

	30 Days Mortality		
	OR	95% CI	*p* Value
AF	2.41	1.38–4.21	0.002
MI	3.19	1.85–5.52	<0.001
CHF	1.41	0.84–2.37	0.18
T2D	0.70	0.40–1.23	0.21
PAD	1.31	0.77–2.25	0.31
Tobacco	3.12	1.82–5.36	<0.001
AVF	0.30	0.16–0.57	<0.001
CVC	3.23	1.76–6.01	<0.001
high-NLR	33.28	13.96–79.36	<0.001
high-MLR	9.46	5.06–17.69	<0.001
high-PLR	3.97	2.30–6.87	<0.001

AF = atrial fibrillation; CHF = chronic heart failure; MI = myocardial infarction; T2D = type 2 diabetes; PAD = peripheral artery disease; AVF = arteriovenous fistula; CVC = central venous catheter; NLR = neutrophil–lymphocyte ratio; MLR = monocyte-to-lymphocyte ratio; PLR = platelet-to-lymphocyte ratio.

## Data Availability

Not applicable.
